# Selective brain penetrable Nurr1 transactivator for treating Parkinson's disease

**DOI:** 10.18632/oncotarget.7191

**Published:** 2016-02-04

**Authors:** Jun Wang, Weina Bi, Wei Zhao, Merina Varghese, Rick J. Koch, Ruth H. Walker, Roshantha A. Chandraratna, Martin E. Sanders, Amanda Janesick, Bruce Blumberg, Libby Ward, Lap Ho, Giulio M. Pasinetti

**Affiliations:** ^1^ Department of Neurology, Icahn School of Medicine at Mount Sinai, New York, NY, USA; ^2^ Geriatric Research, Education and Clinical Center, Bronx, NY, USA; ^3^ Department of Neurology, James J. Peters Veterans Affairs Medical Center, Bronx, NY, USA; ^4^ Io Therapeutics Inc., Santa Ana, CA, USA; ^5^ Departments of Developmental and Cell Biology, and Pharmaceutical Sciences, University of California, Irvine, CA, USA

**Keywords:** brain bioavailable, dopaminergic, nuclear receptor related-1 protein, Parkinson's disease, retinoid X receptor, Gerotarget

## Abstract

Parkinson's disease (PD) is one of the most common movement disorders, and currently there is no effective treatment that can slow disease progression. Preserving and enhancing DA neuron survival is increasingly regarded as the most promising therapeutic strategy for treating PD. IRX4204 is a second generation retinoid X receptor (RXR) agonist that has no cross reactivity with retinoic acid receptors, farnesoid X receptor, liver X receptors or *peroxisome proliferator-activated* receptor PPARγ. We found that IRX4204 promotes the survival and maintenance of nigral dopaminergic (DA) neurons in a dose-dependent manner in primary mesencephalic cultures. Brain bioavailability studies demonstrate that IRX4204 can cross the blood brain barrier and reach the brain at nM concentration. Oral administration of IRX4204 can activate nuclear receptor Nurr1 downstream signaling in the *substantia nigra* (SN) andattenuate neurochemical and motor deficits in a rat model of PD. Our study suggests that IRX4204 represents a novel, potent and selective pharmacological means to activate cellular RXR-Nurr1 signaling and promote SN DA neuron survival in PD prevention and/or treatment.

## INTRODUCTION

Parkinson's disease (PD) is the second most common neurodegenerative disease of aging, which currently affects approximately 1-2% of the world's population over age 65. The number of individuals with PD is expected to double by 2030, affecting up to 9.3 million [1]. PD is associated with increased disability, lower quality of life, increased mortality, and increased healthcare costs across all stages of the disease. The annual economic impact of PD in the United States alone is estimated at $10.8 billion [2].

The molecular mechanisms underlying the disease are unknown, but are likely to involve interactions among genetic and environmental factors. The protein α-synuclein is a central component in PD pathogenesis [3, 4]; α-synuclein aggregation with Lewy body formation in *substantia nigra* DA neurons is a main pathological feature leading to DA neuron cell death [5]. The motor symptoms of PD are believed to begin after 40-60% of the dopamine cells in the substantia nigra (SN) pars compacta are lost [6]. Progression of motor symptoms is related to dopamine cell loss and can be assessed clinically using the Unified Parkinson's Disease Rating Scale part III (UPDRS-3), which is sensitive to treatment related changes. Motor symptoms produce significant disability, worsen quality of life, and advance even when treated optimally with currently available symptomatic therapies.

Current therapies for PD are symptomatic treatments for motor complications of the disease; to date there are no disease modifying drug treatments conclusively shown to be neuroprotective or that slow disease progression [7]. Deep brain stimulation dramatically improves “off” time and dyskinesias, but has no effect on disease progression and often worsens speech and balance [8, 9]. Even cell-based therapies, such as human fetal mesencephalic dopaminergic cells, have not produced sustained benefit, resulting in uncontrollable dyskinesias and Lewy body degeneration of grafted fetal neurons [10]. Clearly, new strategies are needed for disease modifying treatments in PD, including novel mechanisms for decreasing the burden of its pathological substrates.

The loss of midbrain dopaminergic (DA) neurons in the SN, leading to striatal dopamine deficiency, is one of the prominent pathological features of PD. Therefore, preserving or enhancing DA neuron survival provides a therapeutic strategy against PD. There is increasing evidence that activation of the nuclear receptor Retinoid X Receptor (RXR) may protect against PD by providing trophic support for DA neurons [11-13]. For example, activation of RXR has been shown to protect cultured DA neurons from degeneration in an *in vitro* model of PD (6-hydroxy dopamine) and to hypoxia [14]. RXR is known to interact with another orphan nuclear receptor, nuclear receptor related-1 protein (Nurr1), forming RXR-Nurr1 heterodimers [15]. Nurr1 is a transcription factor that is expressed in the embryonic ventral midbrain. In developing DA cells, Nurr1 is required for the expression of several genes important for dopamine synthesis and function [16-20]. *Nurr1* can activate the transcription of tyrosine hydroxylase and enhance the expression of dopamine transporters [21, 22], both of which are significantly affected in PD condition. Therefore, Nurr1 can be a potential target for the treatment of PD. Observations associating Nurr1 mutations with familial Parkinson's disease confirm the importance of Nurr1 in the generation and maintenance of DA cells [23, 24]. Activation of RXR-Nurr1 heterodimers with RXR agonists has been shown have been shown to induce RXR-Nurr1 mediated gene transcription and Nurr1 downstream signaling associated with DA neuron function and protection [15-17, 19, 25]. However, currently available RXR ligands are not necessarily selective for RXR. For example, the promising cancer drug, bexarotene, is also a potent activator of retinoic acid receptors, LXR and PPAR-gamma [26, 27]; the non-selective nature of currently available RXR agonists significantly limits their clinical application [28-33]. In the present study, we show that IRX4204, a novel RXR agonist, specifically binds to RXR and is able to transactivate Nurr1. Most importantly, for the first time, we report that oral administration of IRX4204 prevents PD-type degeneration at molecular, cellular and behavioral levels, following experimental DA lesions in a rat model.

## RESULTS

### IRX4204 is a potent and specific RXR agonist

**Figure 1:**
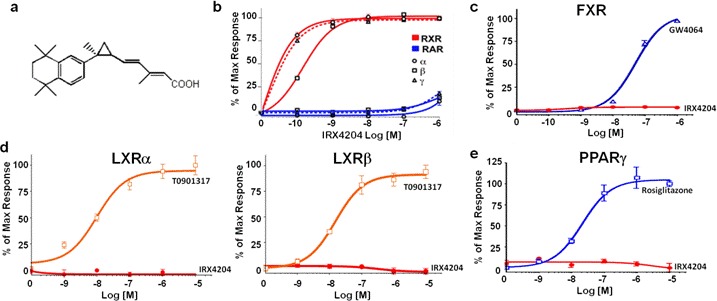
IRX4204 selectively activates RXRs **a.** Structure of IRX4204. **b.**-**e.** Transactivation assay of IRX4204: for receptors RXRα, RXRβ, RXRγ, RARα, RARβ and RARγ **b**.; for farnesoid X receptor FXR **c.**; for liver X receptors LXRα and LXRβ **d.**; for *peroxisome proliferator-activated* receptor PPARγ **e.**; Data are expressed as percent of maximal activity obtained using specific agonists 9-cis-retinoic acid (RXR), retinoic acid (RAR), rosiglitazone (PPARγ), GW4064 (FXR), T0901317 (LXR).

IRX4204 (Figure 1a) is a second generation RXR agonist. We used the receptor transactivation assay to test its specificity. We found that IRX4204 can partially activate RXRs at a concentration of 0.1 nM and fully activates all three RXRs (α, β and γ) at a concentration of 1 nM (Figure 1b). We also found that IRX4204 is thousand times more potent for RXR than retinoic acid receptors (RARα, β and γ, Figure 1b). It does not activate the RXR heterodimers of farnesoid X receptor (FXR), liver X receptors (LXR) α and β, or *peroxisome proliferator-activated* receptor PPARγ, which are responsible for most of the side effects at physiological concentrations (Figure 1c-1e).

### IRX4204 induces RXR-Nurr-1 transactivation and promotes dopaminergic neuron survival in primary VMB neuronal cells

**Figure 2:**
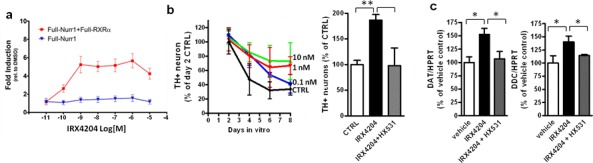
IRX4204 activates Nurr1 and improves DA neuron survival in primary VMB culture **a.** Transactivation assay of IRX4204 on Nurr1 in the presence or absence of RXRα. **b.** IRX4204 promotes DA neuron survival in VMB culture: Time and dose curve of IRX4204 effect on DA neuron survival (left). Promotion of survival by IRX4204 is blocked by RXR antagonist HX531 (right). **c.** IRX4204 promotes Nurr1 downstream DA related gene expression in VMB neurons and induction is blocked by RXR antagonist HX531. All values are expressed as mean ± SEM *p < 0.05, **p < 0.01.

We next tested the effect of IRX4204 on Nurr1 activation using COS7 cells transfected with reporter plasmid and full length Nurr-1 with or without co-transfecting with full-length RXRα plasmid. We found that in the absence of RXRα, the addition of IRX4204 resulted in a base level of reporter gene activation (Figure 2a), while in the presence of RXRα, IRX 4204 potently (EC_50_ <1nM) activated reporter gene transcription in a RXR dependent manner (Figure 2a), indicating that it is an effective activator of RXR/Nurr1 heterodimers. We next investigated the physiological effects of IRX4204 using primary neurons derived from ventral midbrain (VMB). We found that IRX4204 treatment led to increased survival of tyrosine hydroxylase (TH)-positive DA neurons in a dose-dependent manner (Figure 2b, left panel) and that the effects were completely blocked by co-treatment with the RXR antagonist HX531 (Figure 2b, right panel). Moreover, treatment of the VMB neurons with 1nM IRX4204 significantly increased the expression of dopamine transporter (DAT) and dopa-decarboxylase (DDC), and HX531 totally abolished the IRX4204-mediated increased expression of these two genes (Figure 2c).

### Plasma pharmacokinetics, brain bioavailability and activation of Nurr1-downstream gene *in vivo*

Acute and acute-on-chronic plasma pharmacokinetic studies were conducted in rats following oral administration of IRX4204. We found dose-proportional increases in plasma IRX4204 levels (Table 1). However, compared to single acute dosage, plasma concentrations of IRX4204 were substantially lower following repeated dosing, as summarized in Table 1. Both areas under the curve (AUC) and C_max_ values were significantly lower following repeated dosing compared to single acute treatment (Table 1). The increases in C_max_ and AUC_0-24_ were generally dose-proportional on Day 1, but were not consistently proportional to the increases in the dose during the following 4 weeks of treatment. Females had higher C_max_ and AUC_0-24_ values than males on both acute and chronic treatments. For both genders, Week 4 C_max_ and AUC_0-24_ were substantially lower than those observed on Day 1, suggesting changes in disposition of IRX4204 following multiple dose administration in rats. Plasma protein binding, assessed by equilibrium dialysis, revealed that IRX4204 is 99.36±0.07% bound in human plasma and 97.51±0.77% in rat plasma. Our data demonstrate changes in biodisposition of IRX4204 following chronic administration in rats, gender differences in IRX4204 metabolism, and strongly support future pharmacokinetic and pharmacodynamic studies to further understand the metabolism of IRX4204 in healthy and diseased conditions.

**Table 1:** Plasma pharmacokinetics of IRX4204

	IRX4204	1mg/kg/da		3mg/kg/day		10 mg/kg/day	
	Sex	Male	Female	Male	Female	Male	Female
Day 1	Cmax(ng/ml)	58.9	120	198	362	542	974
	Tmax (hours)	0.5	0.5	0.5	0.5	0.25	0.5
	AUC_0-24_ (ng.hr/ml)	140	251	484	975	1489	2437
Week 4	Cmax (ng/ml)	25.7	43.4	54.5	101	77.2	667
	Tmax (hours)	0.25	0.25	0.5	1	0.25	0.25
	AUC_0-24_ (ng.hr/ml)	27.5	136.3	280	344	422	940

Rats were given the indicated dose of IRX4204 once a day through gavage and PK studies were conducted on day 1 and on week 4. n=3 per dose per data point per gender.

**Figure 3:**
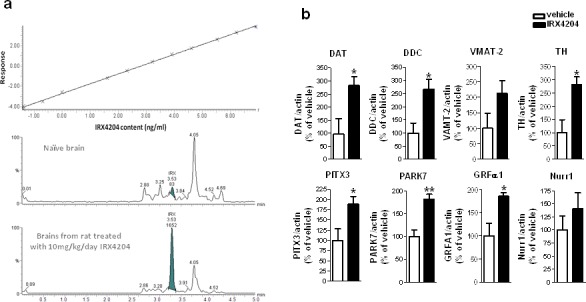
IRX4204 is bioactive in the brains following oral administration and activates Nurr1 downstream genes in the SN **a.** Detection of IRX4204 in the brains: Dose-response calibration curve of IRX4204 detection in the brain matrix with detection limit of 0.25nM (top panel); Representative IRX4204 LC/MS/MS chromatograms from brains of vehicle and IRX4204 treated rats (middle and bottom panel) **b.** Expression of Nurr1 downstream genes in the SN following 7 days oral treatment with 10 mg/kg/day IRX4204. Real-time PCR data are expressed as the percentage of vehicle treated controls.

We next tested whether IRX4204 could cross the blood brain barrier in rats. Following 7 days repeated dosing with 10 mg/kg/day, IRX4204 reached the brain at nM concentrations (11.5±2.9nM), as assessed by LC/MS/MS (Figure 3a). These nM concentrations of IRX4204 in the brain were comparable to those found to promote VMB DA neuron survival (Figure 2b) and to activate RXR/Nurr1 *in vitro* without activating RARs, FXR, LXR and PPARγ (Figure 1b-1e). We also found IRX4204 treatment significantly increased mRNA levels of DA markers in the SN of treated rats, including TH, DAT, DDC, paired-like homeodomain transcription factor 3 (Pitx3), DJ-1(PARK7) and glial cell line-derived neurotrophic factor receptor, GRFα1 (Figure 3b). There was also a trend towards an increase of VMAT-2 (VMAT2) mRNA expression; however, it did not reach statistical significance. Our data suggest that IRX4204 at 10mg/kg/day is sufficient to activate Nurr1-mediated DA neuroprotective mechanisms in the rat brain following oral administration.

### IRX4204 attenuates Parkinson-like phenotypes in a rat model of PD

**Figure 4:**
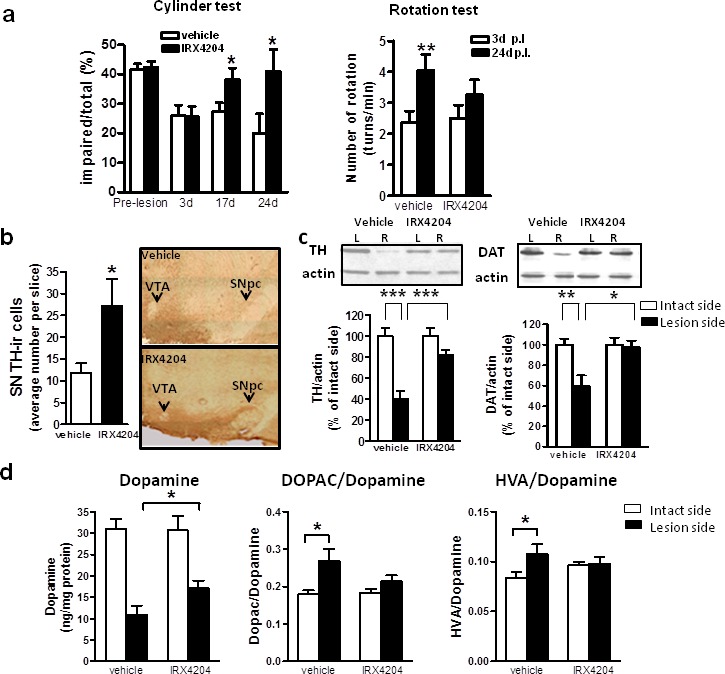
Oral administration of IRX4204 improves motor asymmetry and dopamine neuron loss in a rat model of PD **a**. Behavioral testing following 6-OHDA lesion. Left panel: Cylinder test, data are expressed as percentage of impaired (right) forelimb vs. total (right+left+both) forelimb usage. Right panel: Apomorphine-induced rotation, data are expressed as the number of contralateral rotations per minute. **b**. Quantification of TH-immunoreactive DA neurons in the midbrain SN and representative immunohistochemical images. **c**. Quantification of TH and DAT in the striatum by Western blot analysis and representative western blot images. **d**. Measurements of dopamine concentration in the striatum and the ratios of DOPAC or HVA to dopamine; All values are expressed as mean ± SEM. In a and d, n=15 per group, in b and c, n=8-11 per group; *p<0.5, **p<0.01, ***p<0.001.

Based on this evidence, we tested the preclinical efficacy of IRX4204 at 10mg/kg/day in a rat model of PD. Using standard stereotaxic methodology, all rats were unilaterally injected with 6-hydroxydopamine (6-OHDA) in the right striatum and at three days post-lesion were given either vehicle or 10mg/kg/day IRX4204 for three weeks. Two independent behavioral tests were performed to assess the effects of IRX4204 on the development of hypokinetic motor dysfunction. As expected, all rats developed asymmetric spontaneous use of the limb contralateral to the striatal lesion in the cylinder test three days post-lesion (Figure 4a, left panel). In vehicle-treated animals, impairments persisted at day 17 and day 24 post-lesion (Figure 4a, left panel), while IRX4204 treatment significantly improved spontaneous forelimb use to pre-lesion levels (*p* < 0.05, Figure 4a, left panel), demonstrating that IRX4204 can reverse PD-like motor deficits induced by 6-OHDA lesion. Apomorphine (APO)-induced rotations were assessed as a second independent behavioral measure. During baseline testing three days post-lesion, both groups demonstrated comparable APO-induced contralateral rotations (Figure 4a, right panel). As expected, at day 24 post-lesion, APO-induced rotations increased significantly by almost two-fold compared to baseline among vehicle-treated rats (*p* < 0.01, Figure 4a right panel). In contrast, by day 24 post-lesion for IRX4204-treated rats, the rotations did not differ significantly from baseline (Figure 4a right panel). Collectively, our data demonstrated that IRX4204 is effective in protecting against 6-OHDA induced motor asymmetry.

Following behavioral testing, rats were sacrificed for brain pathology assessment. We found that IRX4204 treatment significantly reduced the loss of TH-immunoreactive neurons in the SN compared to the vehicle-treated group (*p* < 0.05, Figure 4b). Measurements of DAT and TH content in the striatum by Western blot analysis showed that the vehicle-treated group lost ∼40% DAT (59.6%±29.0% of the intact side) and ∼60% TH (40.2±21.6% of the intact side) in the lesioned side compared to the non-lesioned side, while for the IRX4204 group, levels in the lesioned striatum were almost identical to those in the non-lesioned side (Figure 4c). Striatal dopamine loss in the lesioned side was much greater in the vehicle-treated group relative to the IRX4204 group (*p* < 0.01, Figure 4d, left panel). Compared to the non-lesioned side, dopamine turnover, as measured by the ratios of 3,4-dihydroxyphenylacetic acid (DOPAC) or homovanillic acid (HVA) to dopamine in the lesioned side, was increased in the vehicle-treated group, while IRX4204 treatment significantly prevented these increases (*p* < 0.05, Figure 4d, middle and right panel), supporting a neuro-protective role of IRX4204.

## DISCUSSION

Since the discovery of striatal dopamine loss as the main cause of the motor symptoms of parkinsonian, PD therapies have been mainly directed to treat the symptoms focused on the dopamine system. Until now, treatment with L-DOPA to increase the synthesis, storage and release of dopamine in the surviving DA neurons remains the gold standard. Most of the dopatherapies lose their effect with disease progression and patients usually suffer from serious side effects such as involuntary movements [34]. The introduction of dopamine receptor agonists such as ropinirole and pramipexole to reduce the risk of dyskinesia led to the identification of more severe side effects including psychiatric disorders. More recently, surgical therapy targeting the basal ganglia circuitry, such as deep brain stimulation, is also found to be associated with significant side effects. Despite the advances of the current therapeutic approaches targeting the DA system, it is clear that current PD therapies do not reduce the rate or extent of DA neuron loss and have no effect on disease progression. Therefore, neuroprotective interventions able to protect DA neurons and delay disease progression have been actively sought after over the past two decades [7]. The urgent need for novel therapeutic agents for treating PD is further stressed in light of the recent failed phase III creatine clinical trial (http://parkinsontrial.ninds.nih.gov/netpd-LS1-study-termination.htm).

In this study, we characterized the highly selective RXR agonist, IRX4204 in protecting DA neurons.

Nurr1 is a transcription factor critical for DA neuron development and maintenance, and Nurr1 depletion leads to specific DA neuron loss in the ventral midbrain [17, 18, 20]. RXR ligands have been shown to improve DA neuron survival in various *in vitro* models of DA neurodegeneration [14]. The protective benefit of RXR ligands on DA neuron survival is thought to be mediated by signal transduction involving the heterodimeric RXR-Nurr1 nuclear receptor [35, 36]. However despite all this evidence, the development of preclinical and clinical research strategies, aimed at promoting RXR-Nurr1 signaling using RXR agonists, has been hindered by the non-selective nature of the currently available RXR agonists. Most of the RXR agonists have cross-reactivity with RAR, FXR, LXR or PPARγ. The lack of selectivity of RXR agonists have been widely reported for unwanted side effects, which inevitably preclude their development in therapeutic applications for neurodegenerative conditions, including PD [28-33]. Our study, for the first time, strongly supports the characterization of a highly selective RXR agonist, which can activate Nurr1-mediated DA neuroprotective mechanisms in the brain at nM concentration. We also found that oral administration of IRX4204 can significantly increase the expression of molecules that are important for dopamine biosynthesis and metabolism, transport, and for DA neuron survival.

Preserving and enhancing DA neuron survival is the most promising therapeutic strategy for treating PD [37-39]. Our study demonstrates that IRX4204, a highly selective RXR receptor agonist, can transactivate nuclear factor Nurr1 and promote the expression of Nurr1 downstream genes associated with DA neuron function and protection [16, 19, 35, 36]. Moreover, we found that oral administration of IRX4204 attenuates PD-like behavior phenotype and prevent DA neuron loss in a rat model of PD.

We found that IRX4204 treatment can promote the transcription of the receptor for the neurotrophic agent GDNF. This is a very interesting observation, since previous evidence suggests that GDNF failed to elicit DA neuroprotection in a rat model of PD [40], possibly due to α-synuclein mediated neurotoxicity, which was hypothesized to down regulate the gene expression of the GDNF receptor [41]. The failure of past translational clinical application of GDNF in PD can be attributed to this mechanism [42]. Thus, our data demonstrating that oral administration of IRX4204 promotes GDNF receptor expression provides evidence for a strong novel strategy for PD therapy and possibly opens new avenues for combinatory therapy with IRX4204 and GDNF.

In conclusion, our study demonstrates that IRX4204 can penetrate the brain through the blood brain barrier and attenuate PD phenotypes, including neurochemical and motor deficits, in a rat model of PD. Based on the high specificity and potency, the current study suggests that IRX4204 is a highly promising agent for the treatment of PD. Differences in PK between male and female rats are not unusual; however, they can potentially influence the outcome of the treatment efficacy. Future work will continue to assess the pharmacokinetics and pharmacodynamics of IRX4204, which will include both sexes, to determine whether the PK differences lead to pharmacological differences, and to determine the lowest efficacious dose and proper treatment regimen for translational application in PD and possibly combination therapy of IRX4204 and GDNF.

## MATERIALS AND METHODS

### Receptor transactivation assay

For RXR: CV-1 cells were transfected with rCRBPII/RXRE-tk-Luc reporter gene and an expression vector for RXRα, β, or γ. For RAR: CV-1 cells were transfected with ERE-tk-Luc reporter gene and an expression vector for the appropriate ER-RARα, β, or γ. ER-RARs are fusion proteins, composed of the estrogen receptor (ER) DNA binding domain fused to the ligand binding domain of RARs. This system eliminates interference by the low level of endogenous RAR activity in CV1 cells as the readout comes only from the transfected ER-RAR. For PPARγ: CV-1 cells were transfected with 3x(rAOX/DR1)-tk-Luc reporter gene and an expression vector for PPARγ; for FXR: CV-1 cells were transfected with 3x(IBABP/IR1)-tk-Luc reporter gene and vectors for FXR and RXRα; for LXR: CV-1 cells were transfected with 3x(PLTP/LXRE)-tk-Luc reporter gene with vectors for LXRα or LXRβ. For Nurr1: COS7 cells were transfected with 3xNBRE-tk-luc reporter gene and full length Nurr1 with or without full-length RXRα plasmid. Cells were then treated with vehicle or IRX4204 for 20h. Luciferase data were normalized to co-transfected β-gal activity. Luciferase activity is expressed as percent of maximal activity obtained using specific agonists 9-cis-retinoic acid (RXR), retinoic acid (RAR), rosiglitazone (PPARγ), GW4064 (FXR), T0901317 (LXR).

### Primary culture

Ventral midbrain (VMB) from E13.5-14.5 C57BL/6 mouse embryos were dissected, triturated and plated on poly-D-lysine coated 12 well plates or glass coverslips with the serum-free chemically-defined Neurobasal medium, supplemented with 2% B27, 0.5mM L-glutamine, 50I.U./ml penicillin and 50μg/ml streptomycin (Gibco-BRL). For gene expression, cells were treated with 1nM IRX4204 or vehicle with or without RXR antagonist HX531 (100nM) for 16 hours following three days *in vitro* (DIV). For DA neuron survival study, cells were treated with various concentrations of IRX4204 or vehicle following 2 DIV. For each time point, cells were washed once with phosphate buffered saline and fixed with 4% paraformaldehyde for TH immunofluorescent staining. For cell counting, 10 frames were randomly captured from each well using a Leica fluorescent microscope and a minimum of four replicates for each dose and each time point were analyzed for each experiment.

### Animals

Eight week old male Sprague Dawley (SD) rats, weighing 225-250g were housed, one to three per cage, with *ad libitum* food and drinking water and 12:12 hour light:dark cycle. All procedures were approved by the committee for Ethical Conduct in the Care and Use of Laboratory Animals.

### Plasma pharmacokinetics (PK) and plasma protein binding assay

SD male rats were grouped into four groups: vehicle, 1mg/kg/day, 3mg/kg/day and 10mg/kg/day. Rats were treated with the designated dose by daily gavage. Acute plasma PK was conducted on day one and acute-on-chronic plasma PK was conducted on day 24. Pharmacokinetic assessment was conducted by collecting of blood at baseline (prior gavage), 0.25, 0.5, 1, 2, 4, 8, 12 and 24h post gavage. Plasma protein binding to IRX4204 was conducted using the rapid equilibrium dialysis device and K_3_EDTA control plasma of rat, human was spiked with 1ug/ml IRX4204. The spiked plasma was dialyzed at 37°C against an isotonic sodium phosphate buffer to achieve equilibrium. At the end of dialysis, the plasma and buffer level of IRX4204 were quantified by LC/MS/MS.

### IRX4204 content in the brain

Male SD rats underwent daily gavage with 10mg/kg/day for seven days. One hour after the last dose, rats were anesthetized with ketamine/xylazine and transcardially perfused with cold saline. Brains were isolated and homogenized with two volumes of phosphate buffer using ultrasonication. A 100μl sample of the homogenate was transferred to a 1.7ml centrifuge tube and mixed with 20μl of internal standard (100ng/ml of 13-cis retinoic acid-d5 in diluent). Then the sample was deproteinized with 300μl of acetonitrile, centrifuged, and the supernatant was transferred to an autosampler vial for analysis by LC/MS/MS. The calibration standards and the internal standard (13-cis retinoic acid-d5) were diluted in acetonitrile:butylatedhydroxyanisole:ethoxyquin:triethylamine (100:0.05:0.02:0.1, v/w/v/v).

### LC/MS/MS analysis

LC/MS/MS analysis of the samples was conducted by initial separation of the IRX4204 peaks on an HPLC column prior to detection by the mass spectrometer. The LC/MS system was comprised of Waters Acquity Ultra Performance Liquid Chromatography (UPLC) coupled to a XEVO-TQS (Waters). The samples were analyzed by reverse-phase HPLC using a Waters Atlantis T3 column (150 × 2.1mm, 5um). The mobile phase was nebulized using heated nitrogen in a Z-spray source/interface set to electrospray negative ionization mode. The ionized compounds were detected using MS/MS. Standard calibration curve was obtained by spiking known concentrations of IRX4204 to the naïve brain matrix. The detection was linear between 2.5nM to 10nM.

### Quantification of gene expression by real time PCR

Total RNA from VMB neurons or rat substantia nigra were isolated using the RNeasy Mini Kit from Qiagen (Valencia, CA) and 1μg of total RNA was reverse transcribed using SuperScript III first-strand synthesis supermix for qRT-PCR from Invitrogen (Carlsbad, CA) according to the manufacturer's instruction. Quantitative RT-PCR was performed using Maxima SYBR Green master mix (Fermentas) in ABI Prism 7900HT in four replicates with primers amplifying the target genes. Expression level of actin was used as an internal control. Data were normalized using the 2^−ΔΔCt^ method [43]. Levels of target gene mRNAs were expressed relative to those in vehicle control groups and plotted in GraphPad Prism.

### Surgical procedure

All surgical procedures were performed under general anesthesia with ketamine/xylazine. 2μl 6-OHDA (20 mg/ml in 0.02% ascorbic acid) was stereotaxically injected into the right striatum of male SD rats. Lesion coordinates were set according to bregma and dura in mm: ML-3.7; AP-1.0; DV-5.5. The injection rate was 2μl/5 min and the injecting needle was left for 2 minutes after injection to avoid back flow. Apomorphine-induced rotations (see below) were assessed three days post-lesion and rats with ≥ 30 full contralateral rotation/30 minutes were included in the study.

### Drug treatment

Rats were randomly grouped into vehicle control group (*n* = 11) and drug treatment group (*n* = 10). Rats were given 10mg/kg body weight /day IRX4204 by daily intragastric gavage using plastic feeding tubes and the treatment started on post-lesion day four. The control group received DMSO/corn oil (vehicle). The treatment continued for three weeks.

### Behavioral testing

Apomorphine (APO)-induced rotations were performed as described by Fisher et al. [44]. Briefly, immediately after apomorphine injection (0.5 mg/kg body weight), animals were placed in an open area. A camera monitored the animal behavior and computerized software analyzed the number of contralateral and ipsilateral rotations in 30 minutes testing. Rotations were assessed three days post-lesion and after three weeks of treatment (24 days post-lesion). Data were expressed as average contralateral turns per minute.

The cylinder test was used to test for forelimb use asymmetry. Rats to be tested were placed individually in a glass cylinder (21 cm diameter, 34 cm height) and wall exploration was recorded for five minutes. Wall exploration was expressed in terms of the percentage of impaired (right) forelimb vs. total (right+left+both) usage. Paw placement test was performed before the lesion for baseline, three days post lesion and two weeks and three weeks following treatment.

### Immunohistochemistry and cell counting

The caudal portion of the brains were sectioned in the coronal plane on a cryostat at 12 μm. From a random start corresponding to SNpc position, every 12th section was selected -, fixed with 4% paraformaldehyde and processed for tyrosine hydroxylase (TH) staining using mouse monoclonal anti-TH antibody (1:2000, Chemicon) [45] and the Vectastain Elite ABC kit (Vector Lab) according to manufacturer's instruction. TH positive neurons were counted as described [46].

### Measurements of dopamine and metabolites in the striatum by HPLC

The left and right striatum were isolated and homogenized with artificial cerebrospinal fluid and centrifuged at x10,000 g for 15 minutes. Protein content of the supernatant was measured by Bradford assay (Bio-Rad). Dopamine, HVA and DOPAC were measured using an isocratic HPLC system with electrochemical detection, consisting of a pump (ESA model 582 [ESA Chelmsford, MA]), automatic injector (ESA model 542 Autosampler), and a Coulochem III detector (ESA) in conjunction with a guard cell (ESA model 5020) and an analytical cell (ESA model 5014B). Cell potentials were set at +350mV, −150mV, and +220mV respectively for the guard, E1 and E2 cells. A volume of 20μl per sample was injected with a flow rate of 0.6ml/minute, and passed through a 150mm column (ESA model MD-150×3.2) with a particle size of 3μm and pore size of 120Å. Mobile phase consisted of 10% acetonitrile, 90mM NaH_2_PO_4_, 1.7mM 1-octane sulfonic acid, 50mM citric acid, 50μM EDTA; pH≈3). Data were collected and analyzed using the EZStart software (Agilent Technologies, Santa Clara, CA). Standards were run in parallel.

### Immunoblot analysis

Striatum samples were homogenized and boiled in SDS-PAGE sample buffer and separated onto 10% Tris-glycine gels. Western blotting was performed using anti-TH or anti-DAT (Millipore, Billerica, MA), as primary antibodies and enhanced chemiluminescence signals were quantified and normalized with β-actin.

### Overall statistics

In these studies, all values are expressed as mean and standard error of the mean (SEM). Differences between means were analyzed using two-tailed Student t-tests. When comparing three groups, One-way *ANOVA followed by Bonferroni's* post-hoc comparison tests was used. In all analyses, the null hypothesis was rejected at the 0.05 level. All statistical analyses were performed using the Prism Stat program (GraphPad Software, Inc.).
